# Prior Binge Ethanol Exposure Potentiates the Microglial Response in a Model of Alcohol-Induced Neurodegeneration

**DOI:** 10.3390/brainsci6020016

**Published:** 2016-05-26

**Authors:** Simon Alex Marshall, Chelsea Rhea Geil, Kimberly Nixon

**Affiliations:** 1 Department of Psychology & Neuroscience; University of North Carolina-Chapel Hill, Chapel Hill, NC 27599, USA; simon.alexm@unc.edu; 2 Department of Pharmaceutical Sciences, College of Pharmacy, University of Kentucky, Lexington, KY 40536, USA; chelsea.geil@uky.edu

**Keywords:** alcohol, ethanol, microglia, cytokines, TNF-alpha, alcoholism, microglial priming, neurodegeneration

## Abstract

Excessive alcohol consumption results in neurodegeneration which some hypothesize is caused by neuroinflammation. One characteristic of neuroinflammation is microglial activation, but it is now well accepted that microglial activation may be pro- or anti-inflammatory. Recent work indicates that the Majchrowicz model of alcohol-induced neurodegeneration results in anti-inflammatory microglia, while intermittent exposure models with lower doses and blood alcohol levels produce microglia with a pro-inflammatory phenotype. To determine the effect of a repeated binge alcohol exposure, rats received two cycles of the four-day Majchrowicz model. One hemisphere was then used to assess microglia via immunohistochemistry and while the other was used for ELISAs of cytokines and growth factors. A single binge ethanol exposure resulted in low-level of microglial activation; however, a second binge potentiated the microglial response. Specifically, double binge rats had greater OX-42 immunoreactivity, increased ionized calcium-binding adapter molecule 1 (Iba-1+) cells, and upregulated tumor necrosis factor-α (TNF-α) compared with the single binge ethanol group. These data indicate that prior ethanol exposure potentiates a subsequent microglia response, which suggests that the initial exposure to alcohol primes microglia. In summary, repeated ethanol exposure, independent of other immune modulatory events, potentiates microglial activity.

## 1. Introduction

Nearly 14% of the United States population meets the diagnostic criteria for an alcohol use disorder (AUD) in any given year [1]. Excessive alcohol consumption produces neurodegeneration in humans [2,3,4], an effect that has been confirmed in various pre-clinical models [5,6,7,8]. Due to its preventable nature, alcoholism traditionally has not been defined as a neurodegenerative disorder, but chronic, excessive consumption may cause damage in the temporal lobe on par with diseases such as Alzheimer’s [4]. Indeed, alcoholic-related dementia is the second leading cause of dementia in the United States only behind Alzheimer’s disease [9,10]. Even in the absence of dementia, cognitive deficits such as increased impulsivity and impaired executive decision-making are found in many with AUDs [11,12]. Alcohol-induced neurodegeneration and the associated cognitive deficits are thought to be critical factors in the development of AUDs [13,14,15].

Despite the number of reports in human and preclinical models describing the neurotoxic effects of alcohol, the mechanism of how alcohol produces neurodegeneration is unclear [16]. One such mechanism that has recently gained attention is the impact of excessive alcohol consumption on the neuroimmune system, and particularly, microglia [17,18]. Analysis of the brains of human alcoholics suggests that excessive alcohol consumption leads to microglial activation [19,20,21], but whether this activation is the cause or consequence of alcohol-induced neurodegeneration is an active debate [22]. This discussion is due, in part, to a lack of understanding of the effect of alcohol on microglia coupled with the recent appreciation of the role of microglia in both neurodegenerative and regenerative processes [22,23,24,25]. Although microglia have historically been discussed as the phagocytes of the central nervous system (CNS), these cells are far more complex, existing in a continuum of phenotypes or stages of activation [26]. Microglia are constantly surveying the parenchyma in non-pathological conditions; where in response to even a subtle change in their environment, microglia alter their morphological and functional characteristics, a process termed microglial activation [27]. The nomenclature for these stages or phenotypes vary. Terms like M1 and classical activation are applied when microglia have an amoeboid morphology and secrete pro-inflammatory cytokines, whereas M2 and alternative activation are used to describe microglia with bushier ramifications that secrete anti-inflammatory cytokines [26,28]. In neurodegenerative diseases where microglial activation drives neuronal loss, microglia are generally fully or classically activated (*i.e.*, M1 phenotype), secreting pro-inflammatory factors and undergoing uncontrolled phagocytosis [25,29]. How alcohol affects microglia is not well described and appears to vary depending on the model. Most reports of alcohol-induced microglia activation assume that all activated microglia are pro-inflammatory [19,23,30]. However, in the one model with alcohol-induced neurodegeneration, the Majchrowicz four-day binge model, only a low level of activation or alternative (M2) phenotype has been observed [22,24,31].

The variability of microglial phenotypes observed across different AUD models may be due to the pattern of alcohol exposure, specifically intermittent *versus* sustained intoxication. Interestingly, the intermittent exposure models show stronger evidence of pro-inflammatory microglia even with lower doses of ethanol [22,30]. These disparate findings across models led us to question whether the initial hit of alcohol exposure “primes” microglia such that intermittent exposure leads to a potentiated response. Primed microglia have similar morphology and cytokine/growth factor profiles as the M2/alternative microglia, but primed microglial activation is potentiated when subsequent neuroimmunomodulators are applied [28,32,33]. Ethanol’s ability to prime microglia and exacerbate the neuroimmune response to subsequent neuroimmune stimuli is suggested also by the enhanced microglia response to LPS following alcohol exposure [23,34,35]. However, the ability of a second “hit” or insult of ethanol to potentiate the neuroimmune response (independent of peripheral immunomodulators) has not been examined. Therefore, the current study determines whether a second binge ethanol exposure can potentiate the microglia response to binge alcohol exposure. Investigating whether repeated ethanol exposure differentially affects microglia is important considering that the majority of individuals suffering from an AUD drink in a binge pattern that produces periods of high BECs interspersed with periods of withdrawal and abstinence [36,37,38]. Specifically, this study examines both functional and morphological indices of microglial activation in the hippocampus and entorhinal cortex, regions consistently damaged in this model [7,8].

## 2. Materials and Methods

### 2.1. Alcohol Administration Model

A total of 33 adult male Sprague-Dawley rats (Table 1; Charles River Laboratories; Raleigh, NC, USA) were used in these experiments. Procedures performed were approved by the University of Kentucky Institutional Animal Care and Use Committee (protocol #2008-0321, approved 20/6/2008) and conformed to the Guidelines for the Care and Use of Laboratory Animals [39]. Animals weighed approximately 275–300 g at arrival and were pair-housed in a University of Kentucky AALAC accredited vivarium with a 12 h light:dark cycle. Rats were allowed to acclimate to the vivarium for two days followed by three days of handling before any experimentation. Except during the binge periods, animals had *ad libitum* food and water access. Following acclimation, rats underwent a modified version of the Majchrowicz AUD model similar to previously published reports [40,41,42]; however, animals used in this study underwent the Majchrowicz 4-day paradigm twice separated by seven days. Rats were divided into four groups of comparable weights as summarized in Table 1. Briefly, rats were gavaged intragastrically with either ethanol (25% *w/v*) or control diet (isocaloric dextrose) in Vanilla Ensure Plus^®^ (Abbott Laboratories; Chicago, IL, USA) every 8 h. Initially, each rat in an ethanol group received 5 g/kg of ethanol, but subsequent doses were titrated using the individual rat’s behavioral intoxication score on a six-point scale identical to previous reports [40]. Control rats received an average of the volume given to the ethanol group. All rats were then given seven days of recovery with *ad libitum* access to food and water. A seven-day recovery period was chosen because microglial activation is elevated for a week after ethanol exposure [22], and seven days allowed animals to recover from withdrawal and regain body mass lost during the prior binge. Thus, on the 11th day, the Majchrowicz binge model was repeated with rats receiving either ethanol or control diet (Table 1). A separate group had *ad libitum* access to food and water throughout all periods. For all groups, body weights were assessed daily during the binge procedures. The percent difference in weight at the start and end of the 15-day treatment period was calculated.

### 2.2. Blood Ethanol Concentration Determination

To determine blood ethanol concentrations (BECs), tail blood was collected ninety minutes after the seventh session of ethanol dosing during Binge 1 and/or at euthanasia (Binge 2). Bloods were centrifuged for 5 min at 1800 × *g* to separate plasma from red blood cells and immediately stored at −20 °C. BECs were determined from supernatant serum on an AM1 Alcohol Analyser (Analox; London, UK) calibrated against a 300 mg/dL external standard. Each sample was run in triplicate and the average of these runs was calculated and expressed in mg/dL ± SEM.

### 2.3. Tissue Processing

Rats were euthanized within 2–4 h of their final gavage by rapid decapitation. Brains were extracted and dissected into two hemispheres on ice. The left hemisphere was fixed by immersion in 4% paraformaldehyde in phosphate buffer (pH = 7.4) for 2 h, rinsed and stored in phosphate buffered saline at 4 °C until use in immunohistochemical experiments. The right hemisphere was further dissected to remove the hippocampus and entorhinal cortex. Extracted regions were snap frozen on dry ice and stored at −80 °C until use in enzyme linked immunosorbent assays (ELISAs).

### 2.4. Immunohistochemistry

Immunohistochemical procedures were similar to previous reports [22,31]. The left hemisphere was sectioned in a 1:12 series at 40 µm thickness with a vibrating microtome (Leica VT1000S; Wetzlar, Germany) and sections were stored in cryoprotectant at −20 °C. Adjacent series of every 12th section were processed for immunohistochemistry. Briefly, after a series of washes (TBS, pH = 7.5), quenching of endogenous peroxidases (0.6% H_2_O_2_ in TBS) and blocking of nonspecific antibody binding (TBS, 0.1% triton X-100, and 3% horse or goat serum as appropriate), tissue series was incubated overnight in one of the following primary antibodies at 4 °C: mouse anti-OX-42 (1:1000; Serotec MCA275; Raleigh, NC, USA), mouse anti-ED-1 (1:500; Serotec MCA341), mouse anti-OX-6 (1:500; Serotec, MC2687), or rabbit anti-Iba-1 (1:1000; Wako, 019-19741; Richmond, VA, USA). Primaries were chosen for their specificity for microglia phenotypes [26,43]. OX-42 was selected as a marker of microglial activation because it recognizes cluster of differentiation molecule 11b/c (CD11b/c) of complement receptor 3 (CR3), which is constitutively expressed in microglia; however, upregulation of CD11b/c is one of the first indications of microglial activation [44,45,46]. Both ED-1 and OX-6 are selective for more classical forms of microglial activation [26]. ED-1 recognizes the lysosomal membranes of microglia and is thought to be an indication of phagocytic activity [47]. OX-6, however, is an antibody against the major histocompatibility complex-II that elicits T-helper cell activation [26,48]. The Iba-1 antibody was selected because it recognizes a calcium binding protein expressed in all microglia [49]. Sections were incubated in secondary antibody (biotinylated horse anti-mouse, rat adsorbed, or biotinylated goat anti-rabbit, Vector Laboratories, Burlingame, CA, USA), avidin-biotin-peroxidase complex (ABC Elite Kit, Vector Laboratories) and the chromagen, nickel-enhanced 3,3′-diaminobenzidine tetrahydrochloride (Polysciences; Warrington, PA, USA), as previously described [22,31]. Following the final wash, all processed sections were mounted onto glass slides, dried and coverslipped with Cytoseal^®^ (Stephens Scientific, Wayne, NJ, USA).

During quantification, slides were coded to ensure the experimenter was blind to treatment condition. To determine OX-42 immunoreactivity, images of the hippocampus (Bregma −2.50 and −4.00 mm) or entorhinal cortex (Bregma −3.00 and −6.00 mm) were obtained with a 10× objective on an Olympus BX-51 microscope (Olympus, Center Valley, PA, USA) linked to a motorized stage (Prior, Rockland, MA, USA), microcator and DP70 digital camera (Olympus) [50]. OX-42 immunoreactivity was determined by optical density with Visiomorph™ (Visiopharm, Hørsholm, Denmark). Subregions of the hippocampus (dentate gyrus (DG), cornu amonis (CA1 and CA2/3)) and the entorhinal cortex were traced separately and the percent area of OX-42 immunopositive pixels within each region of interest was determined. Immunoreactivity was then normalized to the *ad libitum* control group and expressed as percent of control.

For ED-1 or OX-6 immunohistochemistry, sections were qualitatively assessed in the hippocampus and entorhinal cortex as in past reports [22]. To determine the impact of ethanol on microglia number, Iba-1+ cells were counted within the hippocampus and the entorhinal cortex. Iba-1+ cells within the subregions of the hippocampus were estimated by unbiased stereological methods as previously reported [22,51]. NewCAST™ Stereology software (Visiopharm version 3.6.4.0) coupled to the same Olympus BX-51 microscope system above applied a 70 µm × 70 µm counting frame and cells were randomly sampled using a 20 µm dissector height with 2 µm guard zones within the CA1 (400 µm x,y step length), CA2/3 (250 µm x,y step length), and DG (250 µm x,y step length). Total Iba-1+ cells were calculated using the equation (1):

N = ∑ Q × 1/asf × 1/tsf × 1/ssf(1)

where Q is the number of cells counted, asf is the area sampling fraction, tsf is the thickness sampling fraction, and ssf is the section sampling fraction [52]. Coefficients of error ranged from 0.011 to 0.039 and averaged 0.023 ± 0.001. For the entorhinal cortex, microglia number was determined using a profile counting method [53]. Images of the entorhinal cortex were collected with a 10× objective using a SPOT Advanced™ camera (SPOT Imaging Solutions, Sterling Heights, MI, USA). Iba-1+ cells were quantified in collected images by an automated counting system (Image Pro Plus 6.3; Media Cybernetics, Rockville, MD, USA) and expressed as mean Iba-1+ cells/section ± SEM as previously described [22].

### 2.5. Enzyme Linked Immunosorbent Assay

Hippocampus and entorhinal cortex from the right hemisphere were processed for ELISA as reported previously [22,54]. Briefly, tissues were homogenized in an ice-cold lysis buffer (1 mL of buffer/50 mg of tissue; pH = 7.4), then tumor necrosis factor-α (TNF-α; Invitrogen, #KRC3011C; Camarillo, CA, USA) and interleukin-10 (IL-10; Invitrogen, #KRC0101) cytokine protein was determined via ELISA according to the manufacturer’s instructions. These two cytokines were used to assess pro or anti-inflammatory microglia, respectively [43]. Brain derived neurotrophic factor (BDNF) was measured in the hippocampus (Millipore, #CYT306; Billerica, MA, USA) as the hippocampus is more susceptible to alcohol-induced BDNF dysregulation [55,56]. All samples and standards were run in duplicate. Absorbance was measured at 450 nm on a DXT880 Multimode Detector plate reader (Beckman Coulter; Brea, CA, USA). Cytokine concentrations were normalized to the total protein content as determined by a Pierce BCA Protein Assay Kit (Thermo Scientific; Rockford, IL, USA) and reported as pg/mg of total protein ± SEM.

### 2.6. Statistical Analyses

Data were analyzed and graphed using Prism (version 5.04, GraphPad Software, Inc. La Jolla, CA, USA). Effects were considered significantly different if *p* < 0.05. Behavioral scores were analyzed with a Kruskal-Wallis test. All other analyses used a one-way ANOVA with *post-hoc* Tukey’s test to compare groups if an effect of treatment was observed. Where appropriate, each region of the hippocampus or entorhinal cortex was considered independent and therefore analyzed separately. Correlations were conducted to examine the relationship of microglial markers of activation and the animal model data as well as microglial activation and cytokine concentration. Correlations were only run within the Con/EtOH or EtOH/EtOH group if *post-hoc* analyses showed a significant difference to control groups. Spearman analyses were used for intoxication behavior scores (nonparametric), while Pearson’s analyses were used for all other factors (parametric).

## 3. Results

### 3.1. Animal Treatment Data

For animal model data, each binge period was analyzed independently. For example, BECs from Binge 1 and Binge 2 for the EtOH/EtOH group were analyzed separately. No differences were detected between any groups in either intoxication score (H(3) = 5.60, *p* = 0.07; grand mean = 1.6 ± 0.1) or in BECs (*F*(2,24) = 0.78, *p* = 0.32; grand mean = 399.8 ± 12.4 mg/dL) as shown in Table 2. However, one-way ANOVA revealed differences in the average dose per day (*F*(2,24) = 4.235, *p* = 0.03). A *post-hoc* Tukey’s test indicated that ethanol doses of Binge 2 in the EtOH/EtOH rats were significantly higher than ethanol doses of the single binge (Con/EtOH) rats (Table 2). Body weights were also assessed to determine whether restricted caloric intake affected microglia activation [57,58]. One-way ANOVA indicated that treatment affected weight change (*F*(2,24) = 4.235, *p* = 0.03) (Table 2). A *post-hoc* Tukey’s test showed that the weight change differed between all of the liquid diet groups (Con/Con, Con/EtOH, and EtOH/EtOH) compared with the *ad libitum* group. There was a significant effect of receiving ethanol on weight loss compared with the Con/Con group, but no difference between the Con/EtOH and EtOH/EtOH groups was observed (Table 3).

### 3.2. OX-42 Immunoreactivity Increased by EtOH Exposure

**Figure 1:**
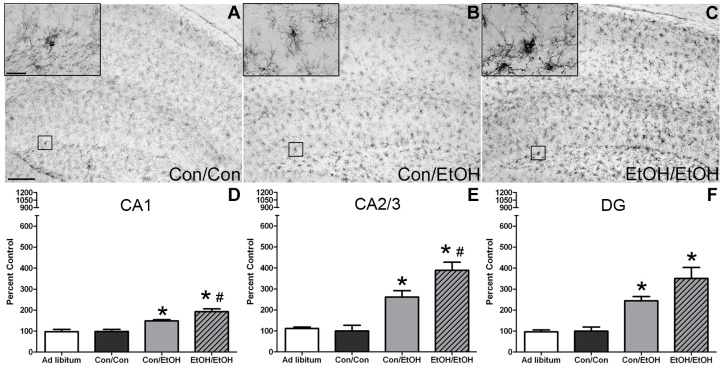
Potentiated Microglial Activation in the Hippocampus by Repeated Ethanol Exposure. OX-42 (CD11b/c) is upregulated in the hippocampus of ethanol-exposed rats as shown in representative photomicrographs of the (**A**–**C**) hippocampal dentate gyrus for (**B**) Con/EtOH and (**C**) EtOH/EtOH groups compared to (**A**) controls. Analysis of OX-42 immunoreactivity indicated that the EtOH/EtOH group had significantly more staining than the Con/EtOH group in the: (**D**) cornu amonis 1 (CA1) and (**E**) cornu amonis 2/3 (CA2/3) regions but not the (F) dentate gyrus (DG). Data expressed as a percentage of *ad libitum* control (not shown). Images were taken at 50× magnification with insets at 600× magnification. Scale bar = 200 µm; inset 30 µm. * *p* < 0.05 compared to *ad libitum* and Con/Con groups; # *p* < 0.05 compared to Con/EtOH.

**Figure 2:**
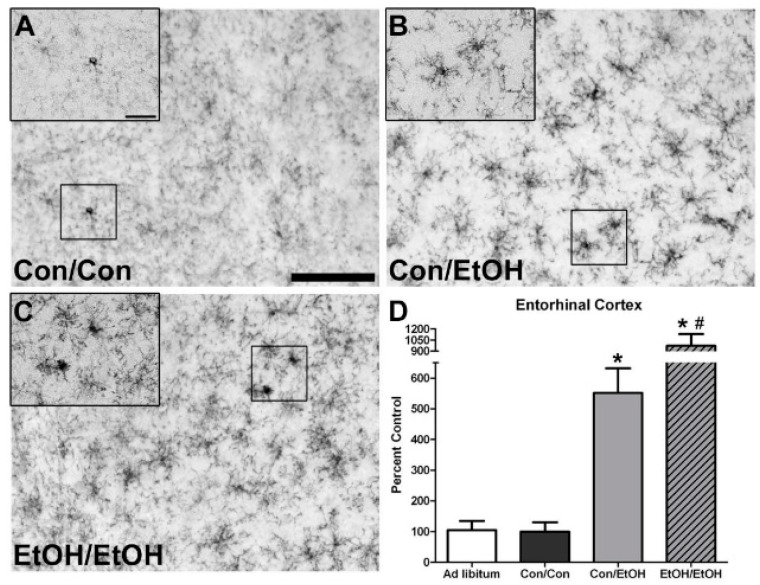
Potentiated Microglial Activation in the Entorhinal Cortex by Repeated Ethanol Exposure. OX-42 (CD11b) is upregulated in the entorhinal cortex of ethanol-exposed rats as shown in representative photomicrographs of the (**A**–**C**) entorhinal cortex for (**B**) Con/EtOH and (**C**) EtOH/EtOH groups compared to (**A**) controls. Analysis of OX-42 immunoreactivity indicated that the EtOH/EtOH group had significantly more positive pixels than the Con/EtOH group in the (**D**) entorhinal cortex. Data expressed as a percentage of *ad libitum* control (not shown). Images were taken at 200× magnification with insets at 600× magnification. Scale bar = 100 µm; inset 30 µm. * *p* < 0.05 compared to *ad libitum* and Con/Con groups; # *p* < 0.05 compared to Con/EtOH.

OX-42 expression was examined to determine whether microglia were further or differentially activated following a second binge exposure. OX-42 positive cells were apparent in all treatment groups, which is consistent with its constitutive expression in all types of microglia [59]; however, there was a visibly distinct increase in immunoreactivity in ethanol treated animals accompanied by an apparent morphological change. Microglia in ethanol animals appeared to have shorter but thickened ramifications compared with the control animals (Figure 1B,C and Figure 2B,C). One-way ANOVAs indicated a significant effect of treatment in the CA1 (*F*(3,29) = 16.81, *p* < 0.0001), CA2/3 (*F*(3,29) = 18.34, *p* < 0.0001), and DG (*F*(3,29) = 14.43, *p* < 0.0001) fields (Figure 1), as well as in the entorhinal cortex (*F*(3,28) = 19.01, *p* < 0.0001) (Figure 2). As expected based on previous data [22], *post-hoc* Tukey’s tests indicated a significant increase in OX-42 density in all ethanol treated groups in all subregions of the hippocampus compared with the control or *ad libitum* groups. Importantly, the EtOH/EtOH group showed greater immunoreactivity than Con/EtOH in all regions analyzed except the DG. Moreover, no difference in OX-42 was observed between *ad libitum* animals and the Con/Con group. Correlations between binge model parameters (intoxication behavior, dose per day, total dose, BEC, percent weight loss) and OX-42 immunoreactivity were run within the EtOH/EtOH and Con/EtOH group, but no significant correlations were observed (Table 4).

### 3.3. Lack of ED-1 or OX-6 Positive Cells

**Figure 3:**
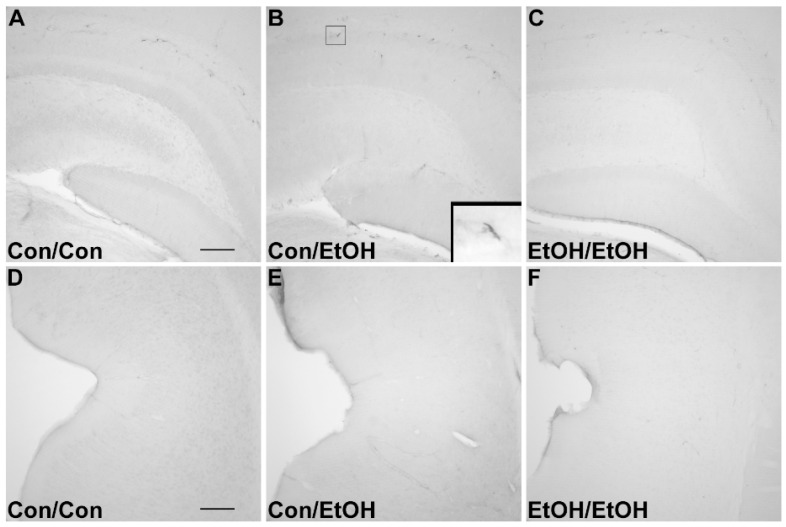
Lack of ED-1 Positive Cells. ED-1 was not visible in the parenchyma of the (**A**–**C**) hippocampus or (**D**–**F**) entorhinal cortex as seen in representative photomicrographs in (**A**,**D**) controls, (**B**,**E**) Con/EtOH (**C**,**F**) or EtOH/EtOH groups. ED-1 positive cells could be seen along the hippocampal fissure and blood vessels as shown in the inset of B. Scale bars = 200 µm.

**Figure 4:**
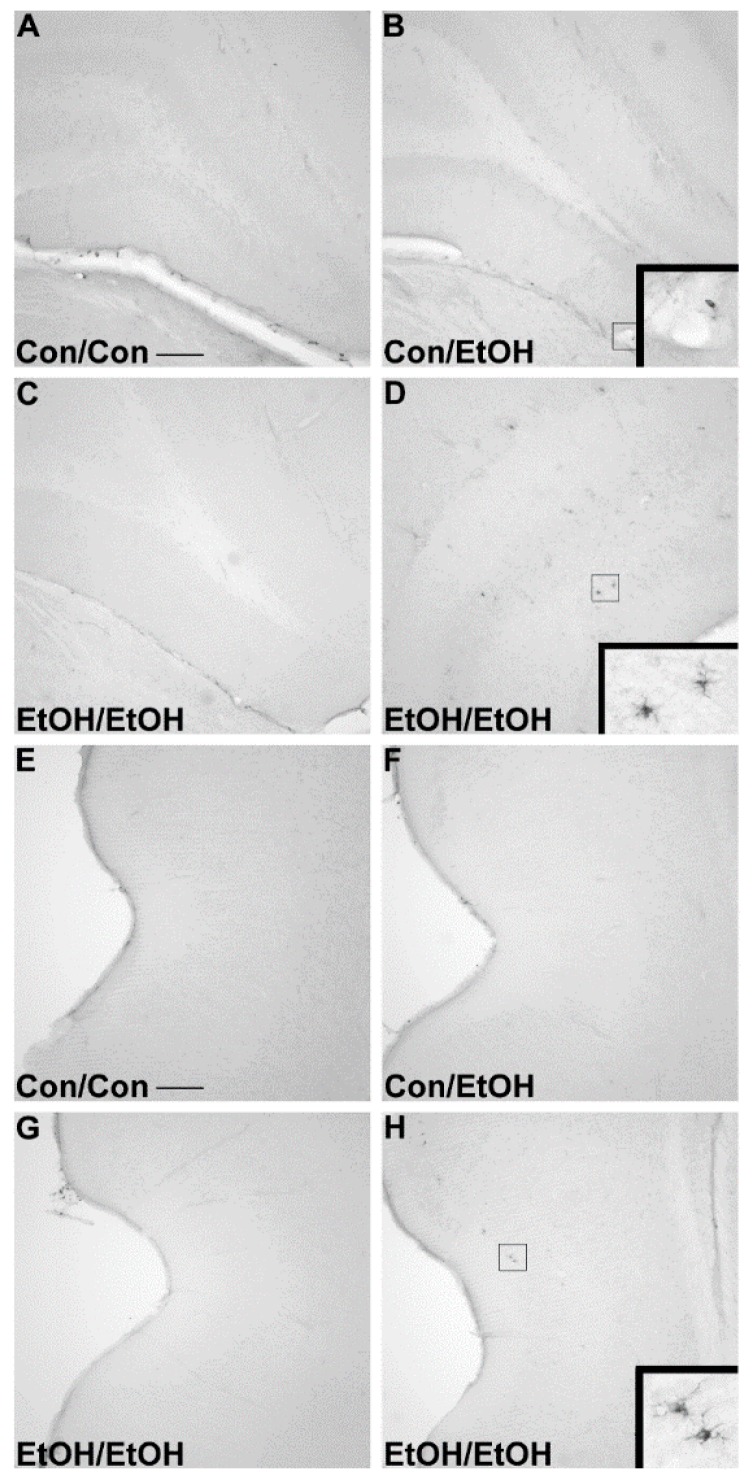
Lack of OX-6 Positive Cells. No OX-6 positive cells were observed regardless of treatment, except in one EtOH/EtOH rat as shown in representative photomicrographs of the (**A**–**C**) hippocampus or (**E**–**H**) entorhinal cortex in (**A**,**E**) controls, (**B**,**F**) Con/EtOH (**C**,**G**) or EtOH/EtOH groups. OX-6 positive cells could be seen along blood vessels as shown in the inset of B. One EtOH/EtOH animal showed upregulation of OX-6 in both the (**D**) hippocampus and (**H**) entorhinal cortex. Scale bars = 200 µm.

The ED-1 antibody was used to identify phagocytic microglia, whereas OX-6 was used to visualize the upregulation of MHC-II [26,29]. No ED-1 (Figure 3) positive cells were observed within the parenchyma of the hippocampus or entorhinal cortex of any animal in any group. No OX-6 (Figure 4) positive cells were observed within the parenchyma of the hippocampus or entorhinal cortex of any group, except for one EtOH/EtOH treated animal. This animal had several OX-6 cells in the more posterior regions of the hippocampus and entorhinal cortex (Figure 4D,H) but was not an outlier for any intoxication parameter including BEC, intoxication behavior, or ethanol dose per day. Interestingly, the morphology of these cells still appeared to be characteristic of the low grade, partial activation state of microglia as they are ramified and not amoeboid [26]. ED-1 and OX-6 positive cells were visible in blood vessels, the hippocampal fissure, and along the meninges in all treatment groups (Figure 3 and Figure 4) similar to that observed previously following binge ethanol exposure [22,60]. Thus, repeated exposure to four-day binge ethanol treatment failed to significantly induce microglia to a phagocytic phenotype or state that expressed MHC-II in the brain parenchyma.

### 3.4. Differential Effects of Treatment on Number of Microglia

**Figure 5:**
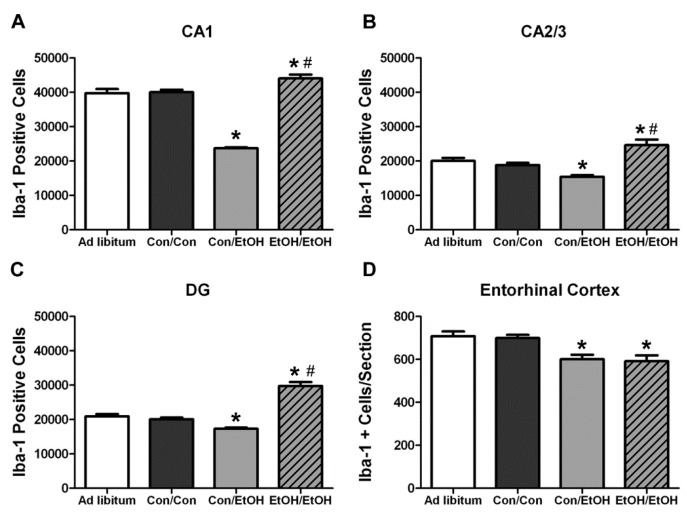
Microglial Cell Counts Differentially Altered by Ethanol Experience. Stereological estimates indicate an increase in the number of microglia in the EtOH/EtOH group in the (**A**) cornu amonis 1 (CA1), (**B**) cornu amonis 2/3 (CA2/3), and (**C**) dentate gyrus (DG) compared with all other groups. However, the number of microglia in the Con/EtOH group was decreased throughout the hippocampus. In the (**D**) entorhinal cortex, microglia were decreased in both the Con/EtOH and EtOH/EtOH groups compared to both the *ad libitum* and Con/Con groups. * *p* < 0.05 compared to *ad libitum* and Con/Con group; # *p* < 0.05 *versus* Con/EtOH.

Stereology and profile counts were used to determine whether repeated ethanol exposure affected the number of microglia during ethanol exposure (Figure 5). One-way ANOVAs indicated a significant effect of treatment in the CA1 (*F*(3,29) = 161.6, *p* < 0.0001), CA2/3 (*F*(3,29) = 17.99, *p* < 0.0001), and DG (*F*(3,29) = 69.98, *p* < 0.0001) fields, as well as in entorhinal cortex (*F*(3,28) = 6.78, *p* = 0.001). *Post-hoc* Tukey’s tests indicated a significant increase in the number of Iba-1+ cells throughout the hippocampus in the EtOH/EtOH group compared with all other groups (Figure 5A–C). However, in the entorhinal cortex microglia cells in the EtOH/EtOH group were decreased compared to the *ad libitum* and control groups but were similar to the number seen in Con/EtOH treated animals (Figure 5D). A *post-hoc* Tukey’s test showed that Con/EtOH rats had decreased Iba-1+ cells in all regions measured as compared to Con/Con and *ad libitum* groups (Figure 5) [61]. Importantly, because the number of microglia can affect immunoreactivity, a correlation between the number of microglia *versus* OX-42 immunoreactivity was run, but no significant relationship was observed.

### 3.5. Increased Pro-Inflammatory Cytokine Expression in EtOH/EtOH Group

**Figure 6:**
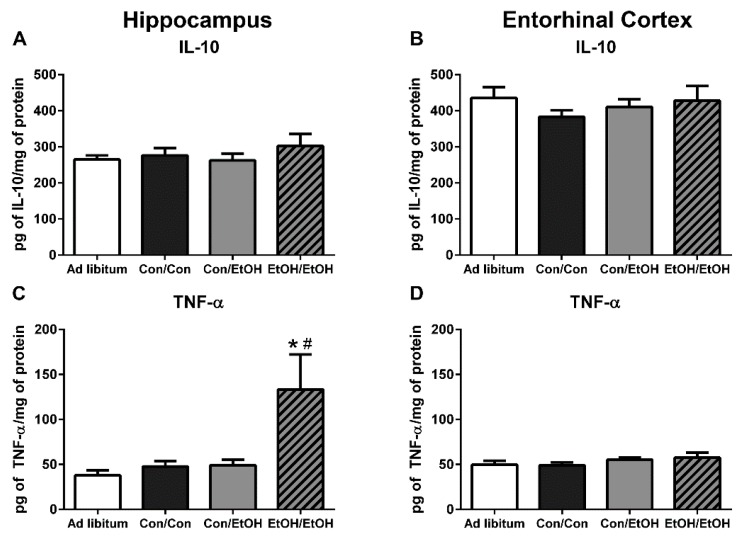
Increased TNF-α in EtOH/EtOH Group. Concentrations of (**A**,**B**) interleukin-10 (IL-10) and (**C**,**D**) tumor necrosis factor-α (TNF-α) were determined by ELISA in both the hippocampus (**A**,**C**) and entorhinal cortex (**B**,**D**). No change in IL-10 was measured in either the hippocampus or the entorhinal cortex, but at least a 2.7-fold increase in TNF-α was measured in the (**C**) hippocampus in the EtOH/EtOH group compared with all other groups. However, no change in TNF-α was seen in the (**D**) entorhinal cortex. * *p* < 0.05 compared to *ad libitum* and Con/Con groups; # *p* < 0.05 compared to Con/EtOH.

**Figure 7:**
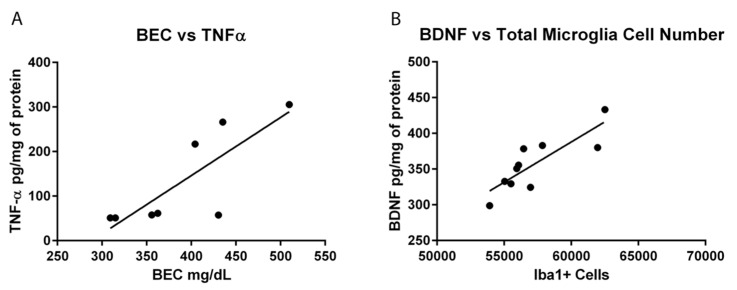
Correlations of Cytokines: (**A**) A positive correlation between blood ethanol concentration (BEC) and tumor necrosis factor-α (TNF-α) concentration. Animals with BECs over 400 mg/dL appear to have an increase in TNF-α. (**B**) A positive correlation between hippocampal estimates of microglia number and brain derived neurotrophic factor (BDNF) concentrations in the Con/EtOH group. A decline in the number of microglia cells correlated with decreases in BDNF concentrations.

ELISAs were used to assess the functional state of microglia, specifically the anti-inflammatory cytokine, IL-10, and the pro-inflammatory cytokine, TNF-α. No changes were seen in IL-10 during intoxication among any groups in either the hippocampus (*F*(3,28) = 0.57, *p* = 0.64) or the entorhinal cortex (*F*(3,24) = 0.50, *p* = 0.69; Figure 6A,B). However, one-way ANOVAs of TNF-α protein concentrations indicated a significant effect of treatment in the hippocampus (*F*(3,28) = 4.658, *p* = 0.009) but not the entorhinal cortex (*F*(3,24) = 0.99, *p* = 0.41). *Post-hoc* Tukey’s tests indicated a significant increase in TNF-α in the hippocampus in the EtOH/EtOH group compared to all other groups (Figure 6C). Correlations of binge parameters *versus* immunohistochemical results were run within the EtOH/EtOH group to further probe the distribution of TNF-α concentrations (Table 5). BECs correlated with TNF-α concentration (P(8) = 0.807, *p* = 0.016; Figure 7).

### 3.6. Differential Effects of Treatment on BDNF Concentrations

**Figure 8:**
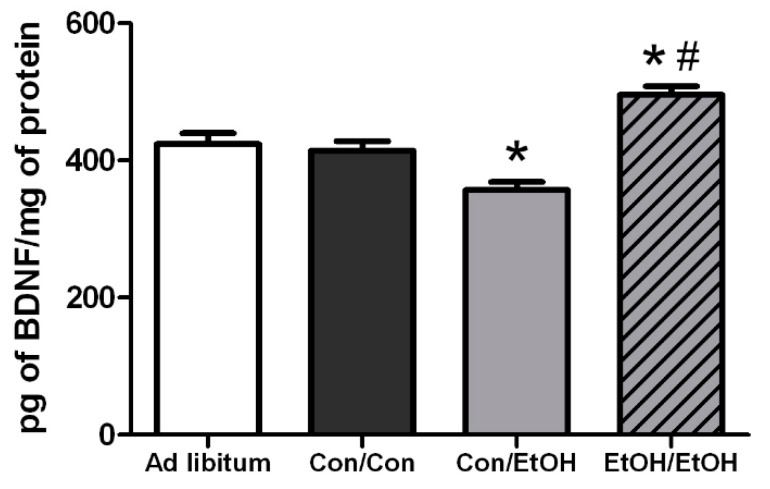
Ethanol Experience-Contingent Effects on BDNF. Concentrations of brain derived neurotrophic factor (BDNF) were determined by ELISA in the hippocampus. BDNF was decreased by approximately 15% in Con/EtOH treated animals compared with Con/Con or *ad libitum* groups but increased by 20% in the EtOH/EtOH group. * *p* < 0.05 *vs. ad libitum* and Con/Con groups; # *p* < 0.05 *vs.* to Con/EtOH.

Hippocampal BDNF concentrations were assessed to see the potential impact of microglia activity because alternative microglia, observed herein, are associated with neurotrophic support [62]. A one-way ANOVA on BDNF concentrations indicated a significant effect of treatment in the hippocampus (*F*(3,28) = 19.00, *p* < 0.0001). *Post-hoc* Tukey’s tests indicated a 20% increase in BDNF concentration in the hippocampus in the EtOH/EtOH compared with all other groups, but Con/EtOH rats had decreased concentrations of BDNF compared to both the Con/Con and *ad libitum* groups (Figure 8). Correlations between binge animal model data as well as markers of microglial activation were run *versus* BDNF concentrations for both the Con/EtOH and EtOH/EtOH groups (Table 5). The estimated total number of microglia (P(10) = 0.835, *p* = 0.003) was correlated to BDNF concentrations only in the Con/EtOH group (Figure 7).

## 4. Discussion

These data collectively indicate that microglia previously activated by alcohol exposure can be further exacerbated by a second alcohol binge. This point was demonstrated by: (a) potentiated OX-42 immunoreactivity; (b) increased microglial number; and (c) increased TNF-α concentration in EtOH/EtOH (double binge) rats compared with Con/EtOH (single binge) rats. The alcohol model used produces a low-grade microglial activation state that is similar to an M2 phenotype [22,31]. However, as the subsequent binge produced more pro-inflammatory-like effects, these alcohol-activated microglia may also be primed. This enhanced response to a second binge aligns with the definition of microglial priming, which is where a stimulus changes microglia to be more susceptible to and over-respond to a second insult [33,63,64]. Primed microglia and/or an exacerbated microglial response could lead to abnormally increased cell death and is a hypothesized etiology of neurodegenerative disorders [28]. Furthermore, given that the majority of individuals with an AUD drink in an episodic binge pattern [38,65], the repeated cycles of binge drinking with periods of withdrawal, and therefore repeated microglial insult, may lead to even more dynamic microglial activation over time.

The first evidence of this potentiated microglia response in the double binge group was increased immunoreactivity to the OX-42 antibody. Increased OX-42 immunoreactivity, which labels CR3, is one of the earliest signs of microglial activation [22,46]. CR3 is associated with cell adhesion necessary for removing pathogens or damaged/dying neurons [45,66,67]. Increased OX-42 staining has been reported in a number of animal models of ethanol exposure [22,68,69,70]. The current study confirms those findings; but furthers that work by showing that a second hit of binge ethanol exposure potentiates OX-42 immunoreactivity. A potentiated increase in OX-42 immunoreactivity, or CR3 density, by ethanol is particularly interesting because CR3 is intimately involved in microglial priming [33]. The increased upregulation of CR3 in the EtOH/EtOH (double binge) rats compared with Con/EtOH (single binge) rats suggests that binge ethanol exposure acts as a priming stimulus to microglia. Morphology, though not specifically quantified, appeared consistent with a low grade/phenotype of activation as cells were ramified and not amoeboid (e.g., Figure 1) [26]. A bushy, ramified microglial morphology is also consistent with that observed in other pathologies that report a primed microglia state [33,64,71]. Furthermore, despite the potentiation of CR3 receptor density, no changes in ED-1 or OX-6 expression were seen following the second binge. The lack of visible ED-1+ or OX-6+ cells concurs with other reports in this model that do not show signs of classical microglial activation following ethanol exposure [22,31,60].

Because multiple endpoints should be measured to understand the phenotype of microglia after insult, functional outputs such as hallmark pro- and anti-inflammatory cytokines were measured to better understand the type of microglial activation associated with a second “hit” of ethanol exposure. No change in the concentration of the hallmark anti-inflammatory cytokine, IL-10, was observed in either ethanol exposure group in the hippocampus or entorhinal cortex. The lack of IL-10 response during intoxication confirms previous findings in this model, although IL-10 is decreased in a mouse AUD model [22,23]. However, upregulation of TNF-α in the hippocampus in the EtOH/EtOH group compared with all other groups suggests that the second binge promoted a pro-inflammatory state. This finding is highly distinct from multiple previous reports using Majchrowicz-like models where no effect of ethanol was observed on TNF-α concentrations [22,24,31] and highlights the impact of repeated ethanol exposure on pro-inflammatory cytokine production and microglial activation. The potentiation of TNF-α expression by the second hit of ethanol, much like the morphological indices, is a common response for microglia that are primed and then hit with a secondary peripheral immune insult [28,64,72]. In fact, alcohol and other drugs of abuse have been shown to prime the TNF-α response to other immune stimulators [23,63], but these finding specifically suggest that alcohol exposure can act as both the priming and secondary stimulus resulting in an increase in TNF-α.

In the Majchrowicz model, microglia loss was observed during the last days of intoxication [61], whereas microglia proliferation occurs after the cessation of alcohol exposure, on the second day of abstinence [31,60]. Therefore, Iba-1+ cell number was assessed to determine how multiple cycles of ethanol affects microglia number. The single ethanol binge (Con/EtOH group) reduced the number of Iba-1+ microglia, in both the hippocampus and entorhinal cortex. Our recent work supports that this reduction is likely due to degeneration of microglia following 4-day binge exposure [61]. Interestingly, the second binge (EtOH/EtOH group) resulted in an increased number of Iba-1+ microglia in the hippocampus compared to either the control group or single binge (Con/EtOH) group. It is plausible that the increase in Iba-1+ cells in the EtOH/EtOH group observed in the hippocampus is due to microglial proliferation at two days following the first binge [22,31,60]. This effect also suggests that ethanol does not significantly reduce these newly proliferated microglial cells. It is of note that the effect of ethanol on microglia number varied by region: in the entorhinal cortex, both the single and double binge resulted in a decrease in the number of microglia consistent with our recent report [61]. The lack of increased Iba-1+ cells in the entorhinal cortex of EtOH/EtOH group is likely related to the finding that microglia neither proliferate dramatically at two days post-binge in the entorhinal cortex nor is there a significant increase in microglia number after seven days;, however, both proliferation and increased Iba-1+ cells have been observed in the hippocampus at this same time point [22,60]. Why microglia proliferate in the hippocampus but not entorhinal cortex after binge ethanol exposure is puzzling. Neurons in the entorhinal cortex degenerate more robustly, peaking at four days of exposure [5,7,8], which is followed by other signs of reactive microgliosis [22]. More studies are necessary to fully understand the dynamic effects of alcohol on microglia number, especially considering the recent discoveries that microglia contribute to synapse refinement and plasticity [25]. These data support the hypothesis that a second binge alcohol exposure exacerbates the microglial response, since an increase in the number of activated microglia would likely result in a potentiated neuroimmune response during the second binge.

Hippocampal BDNF concentrations were determined in order to assess the impact of microglia reactivity and changes in microglia number on the surrounding environment. BDNF plays a pivotal role in neuronal integrity and its dysregulation is associated with neurodegeneration [73]. In the Con/EtOH group, BDNF was decreased, the number of microglia were decreased and there was a significant correlation between the number of microglia and BDNF protein expression. However, in the EtOH/EtOH treated animals, where microglia were more activated and their numbers were increased, a significantly higher BDNF concentration was observed, though this value did not correlate significantly to microglia number. It is possible that the increase in BDNF concentrations is due to cells other than microglia, such as astrocytes, neurons, and other CNS cells secreting BDNF [74]. In addition, the effect of ethanol on BDNF expression is quite complex [75]. Nevertheless, the interplay between the increased cytokine and neurotrophin production observed in the EtOH/EtOH group requires further study to understand its functional implications.

The experimental design to use the same animals for both immunohistochemical and ELISA experiments allowed for a series of correlations to help determine what aspect of ethanol exposure, in this AUD model, was associated with microglial reactivity. OX-42 immunoreactivity did not correlate to average dose per day or to the total dose of ethanol in either the Con/EtOH or EtOH/EtOH groups. This lack of correlation is important as immune modulators such as LPS have dose-dependent responses in microglia reactivity [76]. The lack of correlation between OX-42 and total dose of ethanol suggests that ethanol potentiates the OX-42 response by acting as a secondary stimulus rather than an additive effect of the accumulative dose. Moreover, no relationship between the number of Iba-1+ cells and OX-42 immunoreactivity were observed, supporting that increased OX-42 immunoreactivity was a result of microglial activation and not an artifact of the change in cell number [77]. Correlations were also used to examine the relationship between OX-42 immunoreactivity or Iba-1 cell number and functional indices (cytokine/neurotrophin production). However, neither CR3 receptor (OX-42) upregulation nor Iba-1+ cell number was significantly correlated with TNF-α expression. Interestingly, the bimodal distribution of TNF-α production observed in the EtOH/EtOH group did map on to BECs. Although the mechanism by which BECs are related to TNF-α were not measured, at minimum, this correlation suggests that as BECs increase with repeated exposure, a primed microglial state may cause increased pro-inflammatory cytokines. Finally, in relation to BDNF, only microglia cell number in the Con/EtOH group showed a significant correlation with BDNF concentrations supporting the idea that microglial dysfunction and subsequent loss of trophic factors may contribute to neurodegeneration, especially alcoholic brain damage [61]. Correlations are not being interpreted as causation, but they do provide direction for what aspects of alcohol-exposure impact microglia reactivity leading to a primed microglial state.

Some evidence of classical activation has been observed in other AUD models, an effect that may be attributable to species differences and/or variations in the duration and pattern of exposure [30,69]. While previous reports suggested that the difference in microglia reactivity was due to these aforementioned variations in AUD models, the current data in this report more definitively indicates that it is the repeated insult that may drive the greater microglial response. For example, a model of alcohol exposure with lower total doses of alcohol dispersed over a longer period of time produced more OX-6 positive cells than the exposure used herein, where OX-6 expression may have been an anomaly in a single animal [30]. The appearance of the OX-6+ cells, however, in both models still appeared to be the bushy, ramified morphology associated with a low-level or M2-like activation. Indeed, the only alcohol study, human or animal, where ED-1+ microglia have been observed, is from a study in which rats underwent four cycles of a Majchrowicz-like model with three days between binges. However, the high mortality rate and severe weight loss of rats in that report make interpretations difficult. One interpretation is that microglial activation may have occurred due to the stress of repetitive gavage and/or weight loss [57,78,79,80]. Thus, the current study specifically used a seven day abstinence period to allow rats to recover from four days of intoxication and the significant withdrawal sequelae that occurs in this model [40]. Moreover, because some weight loss is observed in the Majchrowicz model [40], repetitive gavage may be stressful [81], and both of these aspects modulate microglial reactivity [78,82], a group with *ad libitum* access to food and water was included. None of the measures of microglia activation were different between the *ad libitum* group and the Con/Con group despite their slight weight loss and experience with gavage. Moreover, weight loss did not correlate with any measure of microglial activation in animals receiving ethanol.

The potentiated microglia activation seen in this double binge AUD model suggests that the microglial response can be altered by ethanol alone and supports the idea that chronic ethanol exposure can elicit a more pro-inflammatory state than a single bout of binge exposure. The lack of expression of ED-1 and morphology of OX-42+ and Iba-1+ cells support that even with the two binges, cells are not fully or classically activated. However, microglia are “further” down the spectrum towards classical activation than a single binge alone. These data coupled with the lack of evidence for classically activated microglia in human alcoholic brain—whether the markers are not expressed or no one has examined those particular markers—supports that initially microglia activation is likely a consequence of alcoholic neuropathology and not a cause. Whether this increased response causes microglia to over-respond to insult or if it makes the brain more susceptible to ongoing neuroinflammation should be considered in future experiments. In addition, how these effects relate to changes in neurodegeneration, specifically neuronal or volume loss, is an important area for future study. Because microglia have the capacity to maintain low grade activation or a primed state for extensive periods following insult, including alcohol exposure [22,83], the episodic nature of binge drinking would lead to a cycle of repeated priming and over-response in individuals suffering from an AUD [18,38,65]. Understanding the mechanisms that underlie or contribute to alcohol-induced neurodegeneration may provide a novel therapeutic target to ameliorate damage and prevent the downward spiral into an AUD [14,84].

## 5. Conclusions

In summary, these studies present a novel view of the impact of alcohol abuse on microglial activity. Specifically, data presented herein indicate that alcohol causes a shift in microglial phenotypes to a primed state. Although this study focuses on how later bouts of alcohol can exacerbate the microglial response, the implications of an alcohol-induced primed microglial state also extend to how the microglia of alcoholics may respond to infections or other alcohol related immune responses in the peripheral system. This research provides a context in which to consider the implications of microglia on alcohol-induced neurodegeneration and further indicates that targeting the neuroimmune system may alleviate deficits caused by excessive alcohol consumption.

## Figures and Tables

**Table 1:** Experimental Design.

Group	Binge 1 (4 Days)	Recovery (7 Days)	Binge 2 (4 Days)
Con/Con (*n* = 10)	Control Diet		Control Diet
Con/EtOH (*n* = 11)	Control Diet	*Ad libitum* chow	Ethanol Diet
EtOH/EtOH (*n* = 8)	Ethanol Diet		Ethanol Diet
*Ad libitum* (*n* = 4)	N/A		N/A

**Table 2:** Alcohol Model Data.

Group	Intoxication Behavior (0–5 Scale)	Dose (g/kg/day)	BEC (mg/dL)
Con/EtOH (15th Day)	1.8 ± 0.1	9.6 ± 0.2	422.2 ± 21.1
EtOH/EtOH Binge 1 (4th Day)	1.7 ± 0.1	9.9 ± 0.4	378.7 ± 17.7
EtOH/EtOH Binge 2 (15th Day)	1.3 ± 0.2	11.0 ± 0.5 ^#^	390.3 ± 24.0

^#^ *p* < 0.05 compared to Con/EtOH.

**Table 3:** Body Weight.

Group	% Difference
Con/Con (*n* = 10)	+1.0% ± 1.4% ^†^
Con/EtOH (*n* = 11)	−6.6% ± 2.1% *
EtOH/EtOH (*n* = 8)	−8.7% ± 1.7% *
*Ad libitum* (*n* = 4)	+25.2% ± 1.7%

* *p* < 0.05 *vs.* Con/Con and *ad libitum*; ^†^ *p* < 0.05 *vs. ad libitum* only.

**Table 4:** No Correlation between OX-42 and Model Parameters or Microglia Number.

-	Hippocampus		Entorhinal Cortex	
Parameter	Con/EtOH	EtOH/EtOH	Con/EtOH	EtOH/EtOH
Intoxication Behavior	S = 0.433	S = 0.523	S = 0.628	S = 0.371
Dose/Day	*p* = −0.321	*p* = −0.053	*p* = −0.488	*p* = −0.456
Total Dose	*p* = −0.303	*p* = −0.0267	*p* = −0.331	*p* = −0.575
BEC	*p* = 0.424	*p* = −0.572	*p* = −0.082	*p* = 0.032
Percent Weight Loss	*p* = −0.222	*p* = 0.249	*p* = 0.029	*p* = 0.319
Iba-1+ Cells	*p* = 0.161	*p* = 0.539	*p* = −0.136	*p* = 0.357

**Table 5:** Select Hippocampal Cytokine and Growth Factor Correlations.

-	TNF-α		BDNF	
Parameter	Con/EtOH	EtOH/EtOH	Con/EtOH	EtOH/EtOH
Intoxication Behavior	S = 0.451	S = 0.371	S = −0.421	S = 0.216
Dose/Day	*p* = −0.525	*p* = −0.544	*p* = 0.166	*p* = −0.149
Total Dose	*p* = −0.496	*p* = −0.355	*p* = 0.160	*p* = −0.144
BEC	*p* = −0.081	*p* = 0.807 *	*p* = 0.166	*p* = 0.298
Percent Weight Loss	*p* = 0.117	*p* = 0.610	*p* = 0.395	*p* = −0.473
OX-42+ Density	*p* = 0.493	*p* = −0.139	*p* = 0.253	*p* = −0.254
Iba-1+ Cells	*p* = −0.225	*p* = −0.372	*p* = 0.835 *	*p* = 0.224

* *p* < 0.05.

## Abbreviations

The following abbreviations are used in this manuscript:

AALAC	Association for the Assessment and Accreditation of Laboratory Animal Care
ANOVA	Analysis of Variance
AUD	Alcohol Use Disorder
BBB	Blood Brain Barrier
BEC	Blood Ethanol Concentration
BDNF	Brain Derived Neurotrophic Factor
CA	Cornu Amonis
CNS	Central Nervous System
Con	Control
CR3	Complement Receptor 3
DG	Dentate Gyrus
ELISA	Enzyme-Linked ImmunoSorbent Assay
EtOH	Ethanol
Iba-1	Ionized calcium-Binding Adapter molecule 1
IL-10	InterLeukin-10
ip	Intraperitoneal
TBS	Tris-Buffered Saline
TNF-α	Tumor Necrosis Factor alpha
